# MR-guided focused ultrasound thalamotomy modulates cerebello-thalamo-cortical tremor network in essential tremor patients

**DOI:** 10.3389/fneur.2025.1526501

**Published:** 2025-04-22

**Authors:** Li Jiang, Dheeraj Gandhi, Andrew Furman, Howard M. Eisenberg, Paul Fishman, Elias R. Melhem, Rao P. Gullapalli, Jiachen Zhuo

**Affiliations:** ^1^Department of Diagnostic Radiology and Nuclear Medicine, University of Maryland School of Medicine, Baltimore, MD, United States; ^2^Center for Advanced Imaging Research (CAIR), University of Maryland School of Medicine, Baltimore, MD, United States; ^3^Department of Neurology, University of Maryland School of Medicine, Baltimore, MD, United States; ^4^Department of Neurosurgery, University of Maryland School of Medicine, Baltimore, MD, United States

**Keywords:** essential tremor, MRgFUS thalamotomy, resting-state fMRI, cerebello-thalamo-cortical network, tremor network

## Abstract

**Objectives:**

To advance the mechanistic understanding of changes occurring to brain connectivity after successful MR-guided Focused Ultrasound ventral intermediate nucleus (VIM) thalamotomy for essential tremor (ET).

**Methods:**

This retrospective study included fifteen right-handed ET patients, who underwent successful unilateral VIM ablation and experienced improved hand tremor on their dominant hand. Resting-state fMRI scans were conducted both before and 1-year post-treatment for all participants. A seed-based whole brain resting-state functional connectivity (FC) analysis was performed, centering on tremor-related regions within the cerebello-thalamo-cortical (CTC) network, including the left and right ventral intermediate nucleus (VIM), primary motor cortex (M1H), and dentate nucleus (DN). The study examined both the changes in FC and their correlation with clinical outcomes evaluated using the Clinical Rating Scale for Tremor (CRST) at the 1-year post-treatment.

**Results:**

ET patients demonstrated significant tremor improvement at the treated hand, which persisted throughout the 1-year study period. Compared with the baseline, FC of both left VIM and right VIM decreased in precentral gyrus and postcentral gyrus; FC of left M1 hand area increased in premotor cortex and supplemental motor area (SMA); and FC of left DN also increased in premotor cortex, SMA, M1, and anterior cingulate cortex (ACC). Association analysis between changes in left VIM functional connectivity and contralateral hand tremor scores revealed a significant negative correlation in the bilateral precentral gyrus, superior parietal lobule, precuneus, occipital cortex, and middle prefrontal cortex. Conversely, a significant positive correlation was observed in the frontal orbital cortex, right insular cortex, temporal pole, hippocampus, left lingual gyrus, right cerebellar lobules IV/V, left cerebellar lobule VI, and vermis IV/V.

**Conclusion:**

Our findings of altered functional connectivity within the cerebello-thalamo-cortical network, encompassing regions involved in motor, sensory, attention, visual, and visuospatial functions, and its association with hand tremor improvement suggest that targeting functional connectivity abnormalities may be a potential approach for alleviating tremor symptoms in ET patients.

## 1 Introduction

Essential tremor (ET) is a prevalent movement disorder characterized by slowly progressive postural and/or kinetic tremors, typically affecting bilateral upper extremities and hands, although it can also affect other parts of the body (1). The cerebellum-thalamo-cortical (CTC) tremor network includes the cerebellum, thalamus, and motor cortex, all of which play key roles in movement regulation. Functional connectivity (FC) refers to the coordinated activity and communication between different regions of the brain. While traditionally viewed as a disorder of the motor system, emerging research highlights the significant role of aberrant functional connectivity (FC) within the cerebellum-thalamo-cortical (CTC) tremor network in the pathophysiology of ET (2–7).

Magnetic Resonance guided focused ultrasound (MRgFUS) thalamotomy is a recently FDA-approved, minimally invasive treatment option for essential tremor refractory to medication, offering an alternative to more invasive surgical procedures like deep brain stimulation (DBS) or radiofrequency thalamotomy (8–11). This procedure employs high energy high frequency ultrasound beams to lesion a small area of brain tissue within the ventral intermediate nucleus (VIM) of the thalamus, a pivotal relay center for sensory and motor signals between the cerebellum and cerebral cortex (12–20). While short-term and long-term (up to 5 years) clinical trial studies have demonstrated promising outcomes for MRgFUS thalamotomy in tremor alleviation (20–22), there remains a dearth of understanding regarding the functional alterations within the CTC tremor network subsequent to treatment and their potential correlation with clinical tremor amelioration.

In ET, alterations in FC within the CTC network disrupt the finely tuned coordination of motor signals, leading to tremor manifestation (7). Previous studies utilizing resting-state fMRI (rs-fMRI) have shown that MRgFUS thalamotomy modulates functional activity and/or connectivity within this network of ET patients for up to 6 months post-surgery (23–25). One investigation employing fractional amplitude of low-frequency fluctuation (fALFF) revealed increased spontaneous neural activity in the sensorimotor cortex and decreased activity in the posterior cingulate cortex of ET patients at 3 and 6 months post-treatment (23). Another study demonstrated reduced average connections among motor-related areas (precentral gyrus, thalamus, and red nucleus, dentate nucleus), decreased connectivity between substantia nigra and external globus pallidum, and reduced total connection in the thalamus immediately and at 3 months post-treatment (24). Additionally, a third study observed a significant increase in FC between the left thalamus and the caudal part of the dorsal premotor cortex at 3 months post-treatment (25). In addition to the widely established role of the cerebellum and the CTC circuit in the pathogenesis of ET, Kindler's study identified tremor-severity related aberrant functional activity in additional network components 6 months after MRgFUS by using an exploratory data-driven approach independent component analysis (26). Furthermore, analysis of structural connectivity using diffusion tensor imaging (DTI) also revealed short- and long-term (up to 6 months post-surgery) changes in white matter integrity within the CTC network or dentato-rubro-thalamo-cortical (DRTC) pathway in ET patients (27, 28). Thus, different components of the CTC network play crucial roles in tremor oscillation in ET patients, and changes in FC may contribute to tremor alleviation up to 6 months following MRgFUS thalamotomy. It's noteworthy that key brain regions within the CTC network associated with tremor include the dentate nucleus in the cerebellum, the VIM in the thalamus, and the sensorimotor cortex in the cerebral cortex, among others.

In this study, we aimed to investigate the long-term effects, extending up to 1 year, of unilateral MRgFUS thalamotomy on brain FC, employing key brain regions within the CTC network as seeds. These seeds comprise the VIM, the hand area of the primary motor cortex (M1H), and the dentate nucleus (DN). Our study sought to achieve several primary objectives: (1) to evaluate the longitudinal tremor improvement over the 1-year duration following MRgFUS thalamotomy; (2) to investigate the sustained changes in resting-state functional connectivity (FC) within the CTC tremor network following treatment; and (3) to assess the correlation between alterations in FC and corresponding changes in tremor severity. Notably, our investigation extends beyond previous studies by focusing on the prolonged effects of MRgFUS thalamotomy, thus providing valuable insights into the sustained therapeutic impact of this intervention on both tremor symptoms and underlying neural connectivity.

## 2 Methods

### 2.1 Participants

This study was designed as a retrospective analysis of prospectively collected clinical and MRI data from 19 ET patients, who underwent unilateral thalamotomy and MRI scans at the university of Maryland Medical Center. All patients provided written informed consent for the study protocol, which was approved by the Ethics Committee of the University of Maryland. All the experiments were conducted in accordance with guidelines and regulations. The data used for this study was also part of a larger clinical trial, as detailed in (20).

All patients exhibited disabling tremors refractory to medication and met the criteria for MRgFUS thalamotomy for the treatment of refractory tremor as the same described by Elias and colleagues (19). Specifically, six patients exhibited rest tremor in the dominant hand, with 4 having slight tremor (amplitude <0.5 cm), 1 having moderate tremor (0.5–1 cm), and 1 having marked tremor (1–2 cm). Additionally, all patients exhibited postural and action/intention tremor in the dominant hand. Eighteen patients were right-handed, and one was left-handed, according to the Edinburgh Handedness Inventory. MRgFUS thalamotomy was performed on the side of the brain contralateral to the dominant handNo major adverse effects were reported in the available clinical records. To maintain consistency in the treatment approach and ensure that observed effects are attributable specifically to left VIM FUS treatment, the patient who underwent right VIM treatment was excluded from the analysis to mitigate potential confounding factors stemming from differences in treatment location. Additionally, three patients who did not provide rs-fMRI data at all three sessions (baseline, post-24 h, and post-1 year treatment) were excluded from the current analysis. Finally, total of 15 right-handed ET patients who completed successful MRgFUS VIM ablation on the dominant hand side (left hemisphere) and resting-state fMRI (rs-fMRI) scans before and 1-year post-treatment were included in this study.

### 2.2 Clinical outcome assessment

The Clinical Rating Scale for Tremor (CRST) was administered by a neurologist or neurosurgeon specializing in movement disorders at pre-surgery, as well as at 1-, 3-, 6-, and 12-months post-surgery. The assessment consists of three parts: Part A measures tremor at rest, with posture holding, and with action/intention maneuvers for nine body parts (face, tongue, voice, head, trunk, left and right upper extremities, and left and right lower extremities). Part B evaluates kinetic hand tremor, including dominant handwriting, left-hand and right-hand drawing spirals and lines, and pouring. Part C assesses functional disability by evaluating tremor severity during activities such as speaking, eating (feeding), drinking, hygiene, dressing, working, and social activities. A scale from 0 to 4 is used to score each item, with higher scores indicating more severe tremor.

This study aimed to examine the longitudinal changes in kinetic tremor of the upper extremity and hand, with a particular focus on the treated side. We measured hand tremor for the left and right hand (LH and RH) by summing the scores for handwriting, drawing and pouring in part B. Functional disability was assessed by calculating the sum of the scores for all items in part C. The difference in tremor score was calculated by subtracting the post-surgery score from the pre-surgery score, such that a larger positive value indicates greater tremor reduction and greater tremor improvement.

### 2.3 MRgFUS VIM thalamotomy

The ExAblate Neuro System, developed by InSightec in Tirat Carmel, Israel, was utilized for performing a unilateral MR-guided focused ultrasound (MRgFUS) thalamotomy targeting the VIM contralateral to the dominant hand. This surgery was conducted within a 3T MRI scanner, following previously established protocols (20–22). Patients were positioned in a stereotactic head frame coupled to an MRI-compatible ultrasound transducer, with temperature monitored using MR brain thermography. The planning treatment target typically lies approximately ¼ of the distance from the posterior commissure (PC) to the anterior commissure (AC), and 14 to 15 mm from the midline within the AC-PC plane.

Initially, three low-energy sonications generating temperatures around 40°C were applied to refine ultrasound beam adjustment and confirm accurate focusing in three orthogonal planes via magnetic resonance (MR) thermography. Subsequently, 1–2 sonications were delivered with temperatures reaching 45–50°C while adjusting the target location (e.g., adjusting in the z-direction from 0 to 0.2 mm above the AC-PC plane), ensuring slight tremor reduction without off-target effects such as weakness, altered sensation, or speech impairment.

Therapeutic sonications lasting >20 seconds were then administered, gradually escalating power while monitoring target site temperature. Multiple sonications were performed at temperatures of 55–60°C to achieve tissue ablation and the desired clinical effect. Clinical assessments for tremor suppression and any side effects were conducted outside the scanner after each sonication. The procedure continued until sustained therapeutic sonications at this temperature range resulted in optimal tremor reduction (Table 1).

**Table 1:** Characteristics of MRgFUS thalamotomy.

**Patient#**	**Gender**	**Age (year)**	**Treated side**	**Sonication number**	**Sonation beyond 53°C**	**Highest temperature (°C)**	**Highest avg temperature (°C)**	**Max dose area**	**Treated dose volume**	**Lesion volume (mm** ^**3**^ **)**	
										**Post-24 h**	**Post-1 y**
1	M	61	Left	20	0	46.80	43.79	9.57	0.06	55	5
2	M	77	Left	21	9	59.43	55.52	9.57	0.04	75	20
3	M	76	Left	10	7	61.55	56.61	8.37	0.04	72	13
4	F	76	Left	11	7	59.33	56.98	9.57	0.16	86	19
5	M	77	Left	17	5	59.43	55.67	8.37	0.06	73	30
6	M	70	Left	19	7	61.73	56.37	17.95	0.09	86	78
7	M	80	Left	18	5	56.80	53.63	14.36	0.09	26	21
8	M	73	Left	20	13	66.44	58.84	15.55	0.14	215	16
9	M	71	Left	15	3	62.39	57.93	21.54	0.09	185	54
10	M	65	Left	12	7	63.67	60.01	23.93	0.30	137	15
11	M	79	Left	35	21	63.29	59.83	19.14	0.07	201	34
12	M	73	Left	20	6	60.58	57.52	16.75	0.06	239	42
13	M	66	Left	12	7	61.35	57.06	14.36	0.08	95	19
14	M	74	Left	19	13	63.46	58.30	10.77	0.06	110	11
15	M	70	Left	13	6	59.27	55.40	10.77	0.06	261	17
Mean		72.56		17.47	7.73	60.37	56.23	14.04	0.09	14.04	26.27
Stdev	14M/1F	5.41		6.14	4.92	4.43	3.85	5.00	0.07	5.00	19.12
Range		61–80		10–35	0–21	46–66	53–60	8.37–23.93	0.04–0.30	26–239	5–78

The side effects, including those related to speech and gait, were also evaluated following the treatment and during the follow-ups, as described in (20). The clinical findings from the patients in this study were consistent with those reported in the larger trial (20).

### 2.4 MRI Data acquisition

MRI scans were conducted using the same 3T Siemens Trio Tim scanner equipped with 12-channel head coil at the University of Maryland Baltimore at three timepoints: before surgery, as well as 24 h and 1 year after surgery. The MR imaging protocol was the same at all three time points. 3D isotropic T1 MPRAGE images were acquired in the sagittal direction, with imaging parameters: TR = 4,000 ms, TE = 2.96 ms, TI = 1,500 ms, flip angle = 6°, 176 sections per slab, slice thickness = 1 mm, image size = 256 × 256 voxels, and spatial resolution of 1 × 1 × 1 mm^3^. 3D isotropic T2 weighted images were obtained in the axial direction, with imaging parameters: TR = 3,000 ms, TE = 222 ms, flip angle = 120°, slice thickness = 1 mm, image size = 256 × 256, and spatial resolution = 1 × 1 × 1 mm^3^. Resting-state fMRI data was acquired using a T2^*^-weighted single-shot echo planar imaging (EPI) sequence in the axial plane, with imaging parameters of TR = 3,000 ms, TE = 30 ms, flip angle = 90°, slice thickness = 2.7 mm, and in-plane resolution of 2.738 mm × 2.738 mm. A total of 200 volumes were obtained with a total acquisition time of 10 min. To minimize head motion during the scan, patients' heads were secured with foam padding and a comfortable yet firm fixation strap. Additionally, patients were instructed to relax, avoid unnecessary movements, and breathe naturally throughout the scan.

**Figure 1:**
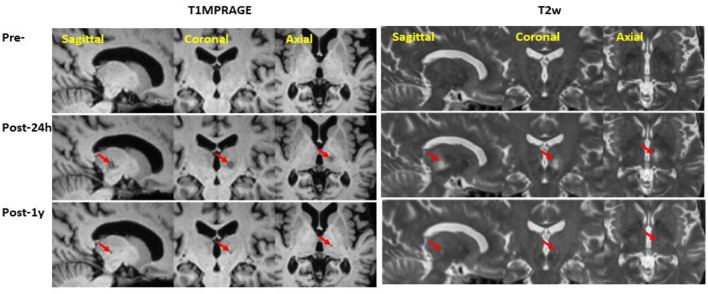
Representative VIM lesion overlapped on T1MPRAGE and T2w at pre-treatment, 24-h, and 1-year post the unilateral MRgFUS thalamotomy.

Figure 1 illustrates a circular ablated lesion in the left ventral intermediate nucleus (VIM) region following MR-guided focused ultrasound (MRgFUS) thalamotomy.

### 2.5 Seed-based functional connectivity analysis

MRI data preprocessing and functional connectivity analysis were performed using the CONN functional connectivity toolbox v15.h (http://www.nitrc.org/projects/conn). First, T1-MPRAGE structural images underwent non-linear registration and normalization to the MNI average152 template image with a 1 × 1 × 1 mm^3^ resolution. Preprocessing of rs-fMRI data included slice-timing correction, realignment, detection of motion outliers, and direct normalization to the MNI template space, resulting in a resolution of 2 × 2 × 2 mm^3^. Spatial smoothing was applied using an 8 mm full-width half maximum (FWHM) Gaussian Kernel. To eliminate potential confounds associated with motion and non-neuronal contributions to the BOLD signal, the five principal components of BOLD signal from the WM and CSF, as well as 12 regressors for motion parameters and their first derivatives and motion outlier scrubbing (>2.5 mm), were regressed from the data. Temporal filtering was performed with a band-pass filter of 0.008~0.09 Hz and linear detrending were applied after regression to further account for nuisance variables.

To examine changes in functional connectivity, we performed seed-based functional connectivity analysis with the hand area of the primary motor cortex (M1), the ventral intermediate nucleus (VIM), and the dentate nucleus (DN) of the cerebellum serving as seeds. The individual VIM ablation was manually delineated using fMRI data from the 12-month post-treatment visit. To generate a probability map of the VIM lesion, the binary lesion masks from all subjects were combined, with voxel values representing the frequency of lesion overlap. The VIM seed coordinate was then identified as the region with the highest lesion occurrence across subjects. A 3 mm spherical region was created around this coordinate as the seed. Finally, the seed location was visually inspected for all subjects to ensure it was properly aligned with the lesion site. The coordinates of the left VIM were estimated as (−13, −19, 0) mm. The contralateral seed region (right VIM) was obtained by reflecting the left VIM seed across the midline of MNI space, i.e., (13, −19, 0) mm. The MNI coordinates of M1H were based on an independent study of actual hand movements (25, 26). The left and right M1H were located at (+/– 41, −20, 62) mm. The left and right dentate nucleus (DN) were located at (±14, −58, −36) mm. Seed masks were created by placing a 3 mm sphere for VIM and 5 mm sphere for other seeds at the estimated coordinates.

For each seed, the BOLD time series was obtained by averaging the signal across all voxels within the seed. Correlation maps were then generated for each seed by calculating the Pearson correlation coefficient between the seed time series and the time series of all other voxels in the whole brain. To account for violations of normality in the resulting correlation coefficient distribution, a Fisher-z transform was applied separately to the correlation coefficient values belonging to each individual seed analysis. Correlation transformation was applied to each patient at each time point (baseline and post-1 year) separately.

### 2.6 Voxel-wise association between tremor score and left VIM functional connectivity

A general linear regression model was used to estimate the voxel-wised association between changes in FC of the left VIM seed (post-1 year minus pre) and contralateral hand tremor improvement (pre minus post-1 year). Age and sex was included as covariates.

### 2.7 Statistical analysis

A one-way repeated measures ANOVA was conducted to evaluate longitudinal changes in CRST scores across all time points (pre-treatment, 1 month, 3 months, 6 months, and 12 months post-surgery) using IBM SPSS 21.0 (IBM Corporation, Chicago, IL). Bonferroni *post hoc* analysis was applied for multiple comparisons, with a significance level set at *p* < 0.05.

A paired *t*-test was conducted to compare changes in FC between baseline and 12 months post-surgery, while controlling for age and gender. The significance threshold was set at an uncorrected voxel-wise *p* < 0.01, with cluster-wise FDR correction at *p* < 0.05 for multiple comparisons.

Significant associations between FC and clinical changes were identified using voxel-wise uncorrected *p* < 0.01, with cluster-wise FDR correction at *p* < 0.05 for multiple comparison.

## 3 Results

### 3.1 Patient demographics and clinical data at baseline

Table 2 summarizes the demographic information and clinical data for ET patients in this longitudinal study before unilateral MRgFUS thalamotomy. The 15 ET patients (age: 72.74 ± 5.56 years; 14 Male/1 Female) were all right-handed. Fourteen out of the 15 ET patients demonstrated dominant tremor at the right upper extremity and right hand.

**Table 2:** Demographics of ET patients and clinical characteristics at baseline.

**Measure**	**ET**
N	15
Age (year)	72.74 ± 5.56
Gender	14 Male/1 Female
Handness	15 right handed
**Upper extremity tremor**	
Right upper extremity tremor score	5.57 ± 1.09
Left upper extremity tremor score	4.71 ± 1.86
Right > Left (N)	9
Right = Left (N)	5
Right <Left (N)	1
**Hand tremor**	
Right hand tremor score	11.57 ± 3.50
Left hand tremor score	9.21 ± 4.00
Right > Left (N)	8
Right = Left (N)	6
Right <Left (N)	1

### 3.2 Hand tremor and functional disability following MRgFUS thalamotomy

**Figure 2:**
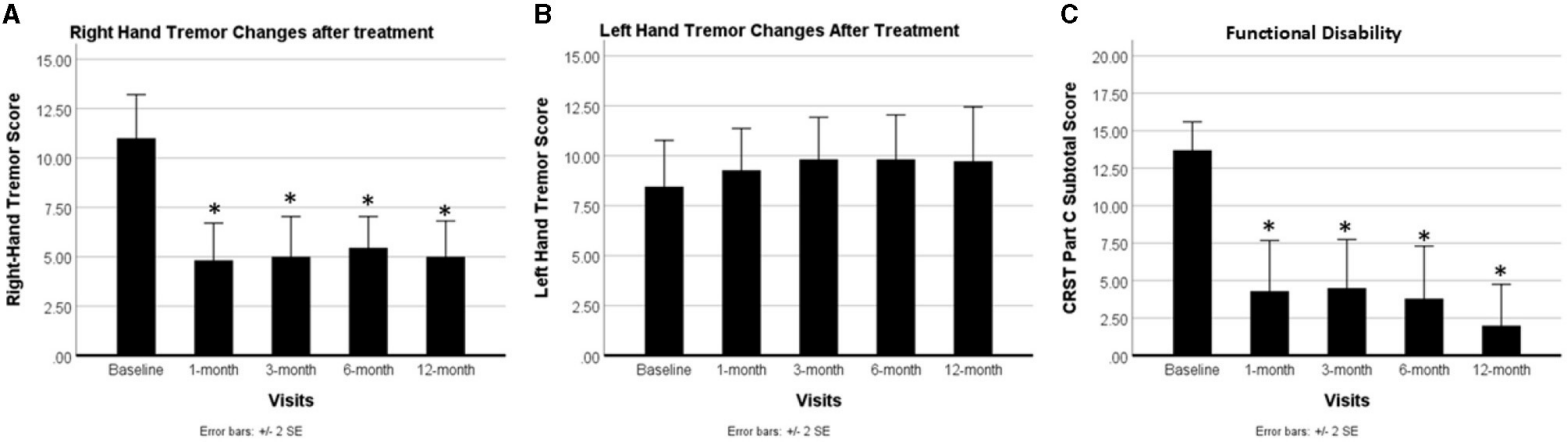
Improvement in hand tremor and functional disability following MRgFUS thalamotomy treatment. **(A)** Right-hand tremor score; **(B)** left-hand tremor score; and **(C)** functional disability score. The error bars represent the standard error of the mean (SEM). After thalamotomy treatment, the tremor was significantly reduced in the right hand (treated) and this reduction was maintained throughout the 12-month study period, while the tremor score in the left hand (untreated) showed no change. The functional disability for patients significantly improved following MRgFUS thalamotomy and this improvement was sustained throughout the 12-month study period. Asterisk (*) indicates a significant difference between baseline and post-treatment time points at 1, 3, 6, and 12 months.

Figure 2 presents the changes in hand tremor and functional disability across five longitudinal time points: baseline, 1-month, 3-month, 6-month, and 12-month post-treatment. Repeated measures ANOVA with Greenhouse-Geisser correction identified significant alterations in right hand tremor (treated hand) and functional disability across all five time points [*F*_(4)_ = 10.54, *p* = 1.33e-6]. *Post hoc* tests with Bonferroni correction revealed a noteworthy decrease in treated hand tremor immediately after surgery, with this reduction persisting throughout the study period (baseline: 11.33 ± 3.498, post-surgery 1-month: 4.79 ± 2.778, 3-month: 5.71 ± 3.561, 6-month: 5.85 ± 3.051, and 1-year: 5.08 ± 2.875). No significant reduction was observed over time for left hand tremor (untreated hand); if anything, there was a slight increase in left hand tremor.

Functional disability also demonstrated improvement immediately after treatment, with this effect persisting over the study duration (baseline: 13.67 ± 3.266, post-surgery 1-month: 3.71 ± 4.548; 3-month: 4.29 ± 4.393; 6-month: 3.36 ± 4.830; and 12-month: 2.00 ± 4.346).

### 3.3 FC of VIM at baseline

**Figure 3:**
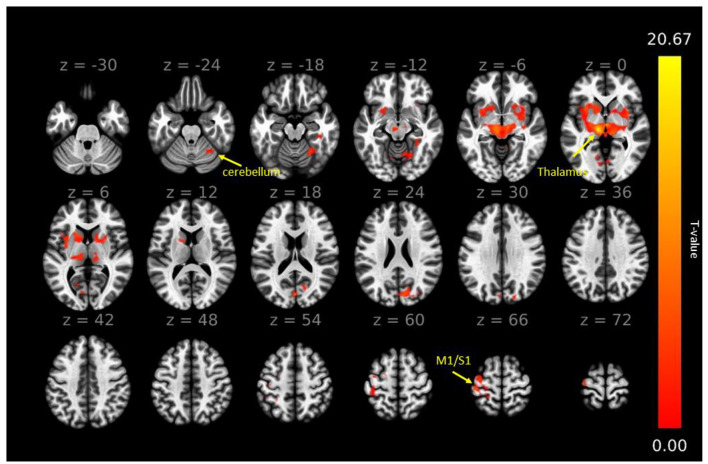
The functional connectivity map of left VIM before the MRgFUS thalamotomy. The map shows widespread functional connectivity within the cerebello-thalamo-cortical tremor network and beyond. The right VIM exhibited a similar functional connectivity pattern (not shown). The significant level was defined as voxel-wise uncorrected *p* < 0.0001 and multiple comparison with cluster-wise corrected FDR *p* < 0.05. The color bar represents the T-value, with red-yellow colors indicating positive correlation. M1, primary motor cortex; S1, primary somatosensory cortex.

As illustrated in Figure 3, prior to surgery the left ventral intermediate nucleus (VIM), demonstrated extensive FC with several brain regions within and beyond the CTC network. Specifically, the left VIM exhibited significant FC with: (1) Cortical regions, encompassing the left primary motor cortex (M1 or PreCG), left primary somatosensory cortex (S1 or PostCG), left superior parietal lobe (SPL), left superior frontal gyrus (SFG), left and right cuneal cortex, and right superior lateral occipital cortex (sLOC), as well as the bilateral insular cortex (IC). (2) Subcortical regions, including the bilateral thalamus, putamen, caudate, pallidum, and brainstem. (3) Cerebellar motor regions, such as the right cerebellum lobular regions IV/V, VI, and vermis regions IV/V. The right VIM exhibited a similar FC pattern to that of the left VIM (data not shown). This observation guided us in selecting seeds for FC analysis within the CTC network, particularly focusing on regions associated with motor function.

### 3.4 FC changes in cerebello-thalamo-cortical network at 1-year post-MRgFUS thalamotomy

**Figure 4:**
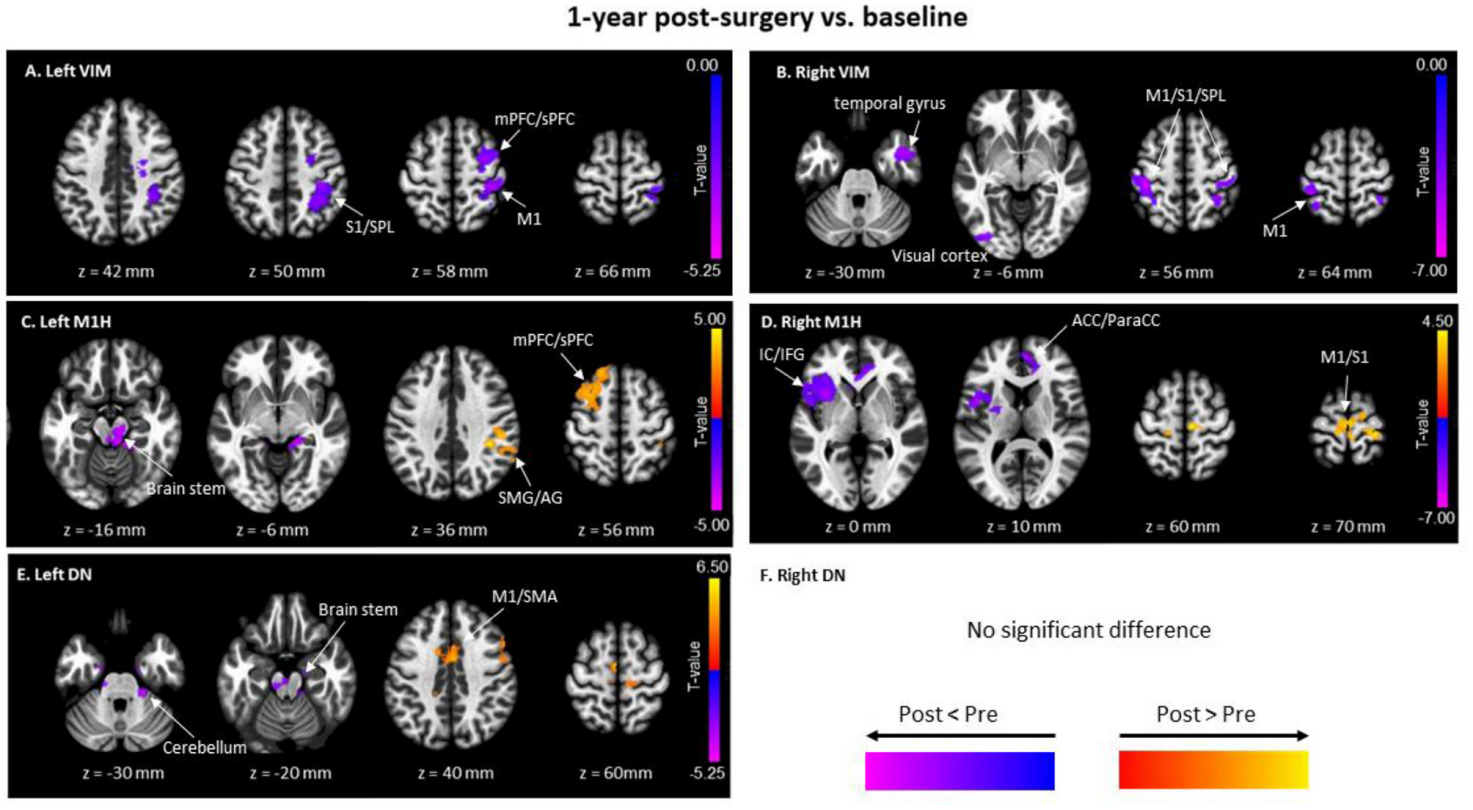
Significant clusters of functional connectivity (FC) differences between baseline and 1-year post-surgery with seeds of **(A)** Left VIM; **(B)** Right VIM; **(C)** Left M1H (Hand Area in Primary Motor Cortex); **(D)** Right M1H; **(E)** Left DN (Dentate Nucleus); and **(F)** Right DN. Pairwise *t-*tests were performed. Red-yellow colors show FC at 1-year post-surgery greater than baseline, and blue-purple; colors represent FC at 1-year post-surgery less than baseline. SMA, supramarginal gyrus; M1, Primary motor cortex; S1, somatosensory cortex; SPL, superior Parietal Lobe; mPFC, middle prefrontal cortex; sPFC, superior prefrontal cortex; SMG, supramarginal gyrus; AG, angular gyrus; ACC, anterior cingulate gyrus; ParaCC, paracingulate gyrus.

Figures 4A, B depict the FC alterations of the left VIM (treated site) and right VIM (non-treated site). The left VIM exhibited a significant decrease in FC with contralateral cortical brain regions including the right precentral gyrus, right postcentral gyrus, superior parietal lobe, middle frontal gyrus, and superior frontal gyrus. Similarly, FC of the right VIM significantly decreased with bilateral precentral gyrus, postcentral gyrus, contralateral occipital fusiform gyrus and inferior occipital cortex, and right middle temporal gyrus and temporal pole.

Figures 4C, D show changes in FC of the left (left M1H) and right (right M1H) hand areas of the primary motor cortex. Compared to baseline, the FC of left M1H significantly increased in the ipsilateral superior frontal gyrus and middle frontal gyrus, the contralateral postcentral gyrus, supramarginal gyrus, and angular gyrus. Additionally, FC of the left M1H decreased in the brainstem, contralateral lingual gyrus, and contralateral cerebellar III and bilateral cerebellar IV/V (voxel-wise uncorrected *p* < 0.009 and cluster-wise FDR *p* < 0.05 for multiple comparisons). The right M1H exhibited enhanced FC in several cortical regions of the sensorimotor network, including bilateral precentral gyrus and supplemental motor area. Conversely, right M1H showed lower FC in frontal lobe regions, including the contralateral insular cortex, frontal operculum, central operculum, frontal orbital cortex, inferior frontal gyrus at opercular part, inferior gyrus at triangular part, and temporal pole, as well as the anterior cingulate gyrus and bilateral paracingulate gyrus.

Only the FC of the left dentate nucleus (DN) showed significant alteration following MRgFUS surgery when compared with baseline, whereas no changes were observed in the FC of the right DN (Figure 4F). Figure 4E illustrates that the FC of the left DN significantly increased with bilateral precentral gyrus, supplemental motor area, premotor area, inferior frontal gyrus, and the anterior cingulate gyrus. Reduced FC was observed between the left DN and the brainstem, right anterior parahippocampus, and right cerebellum lobular III, IV/V (voxel-wise uncorrected *p* < 0.009 and cluster-wise FDR *p* < 0.05 for multiple comparisons).

### 3.5 Correlation between VIM functional connectivity and hand tremor severity changes at 1-year post-MRgFUS thalamotomy

**Figure 5:**
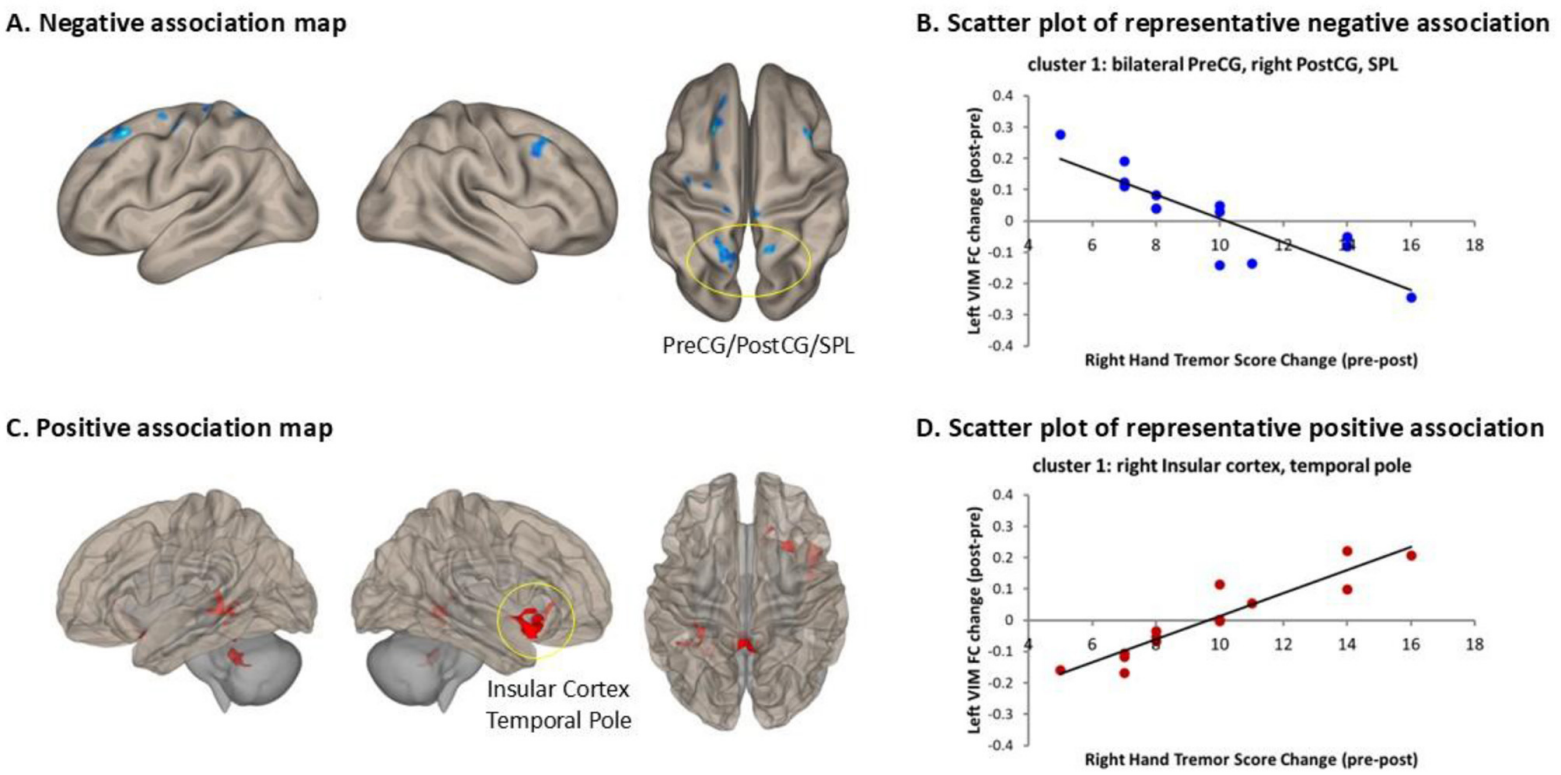
Significant associations between changes in right-hand tremor scores (pre-post) and left VIM FC changes (post-pre). **(A)** Clusters showing significant negative correlations; **(B)** representative scatter plot illustrating the strongest negative correlation in the cluster of bilateral PreCG, left PostCG, and left SPL [cluster coordinates: (−4, −30, 58), cluster size = 421 voxels, cluster p-FDR = 0.000004]; **(C)** clusters showing significant positive correlations; **(D)** representative scatter plot illustrating the strongest positive correlation in the cluster of the insular cortex and temporal pole [cluster coordinates: (46, 12, −16), cluster size = 359 voxels, cluster p-FDR = 0.000033]. PreCG, Precentral Gyrus; PostCG, Postcentral Gyrus; SPL, Superior Parietal Lobule.

Figure 5 presents the significant associations between changes in right-hand tremor scores (pre-post) and left VIM FC changes (post-pre), along with representative scatter plots illustrating the strongest correlations.

Figure 5A displays brain regions showing significant negative correlations, with clusters encompassing the right middle frontal gyrus (MidFG), precentral gyrus (PreCG), postcentral gyrus (PostCG), superior parietal lobule (SPL), as well as the left superior frontal gyrus (SFG), MidFG, PreCG, superior lateral occipital cortex (sLOC), and precuneus. Figure 5B presents a representative scatter plot illustrating the strongest negative correlation within a cluster covering the bilateral PreCG, left PostCG, and left SPL [cluster coordinates: (−4, −30, 58), cluster size = 421 voxels, cluster p-FDR = 0.000004].

Figure 5C shows brain regions with significant positive correlations, including clusters in the right insular cortex, right temporal pole, vermis IV/V, left and right cerebellar lobules IV/V, left cerebellar lobules VI/VIII, and the left hippocampus. Figure 5D presents the representative scatter plot illustrating the strongest positive correlation, located in a cluster covering the right insular cortex and right temporal pole [cluster coordinates: (46, 12, −16), cluster size = 359 voxels, cluster p-FDR = 0.000033].

Detailed statistical information and cluster locations are provided in Supplementary Table 1, and scatter plots of additional clusters are shown in Supplementary Figure 1.

Additionally, no significant correlations were observed between changes in left VIM FC and changes in functional disability scores from CRST Part C.

## 4 Discussion

The findings from our study demonstrate a significant improvement in tremor severity following MRgFUS thalamotomy treatment, as assessed using the CRST. Throughout the 1-year study period, tremor amplitude and functional disability were notably reduced in the treated hand, indicating the effectiveness of MRgFUS thalamotomy in alleviating tremor symptoms. Importantly, the observed improvement in tremor severity is consistent with the outcomes reported in previous clinical trials, thereby reinforcing the efficacy of MRgFUS thalamotomy as a viable therapeutic option for ET patients (20–22). The maintenance of tremor reduction over the 1-year duration underscores the durability of treatment effects and highlights the potential long-term benefits associated with MRgFUS thalamotomy. These findings, corroborated by objective assessments using the CRST, contribute to the growing body of evidence supporting the sustained efficacy of MRgFUS thalamotomy in tremor management and provide valuable insights for clinicians and patients alike.

We observed suppressed FC of the left VIM in sensorimotor cortical regions, including the primary motor cortex and primary somatosensory cortex. The primary motor cortex, being a direct cortical projection of the VIM, plays a pivotal role in tremor generation and increased VIM-related FC was observed in the bilateral primary and supplementary motor cortex in ET patients (29). Additionally, we found suppressed FC between the left VIM and the fronto-parietal network, which serves as a central hub in the brain for coordinating high-order cognitive functions such as attention, execution, and motor planning. The disrupted FC between the left VIM and both the sensorimotor and fronto-parietal networks may directly contribute to tremor reduction following MRgFUS treatment.

Similarly, FC of the right VIM, the intact counterpart of the left VIM, also decreased with the sensorimotor cortical network, bilateral primary motor cortex, and primary somatosensory cortex. The observed changes in FC of the right VIM parallel those of the left VIM, suggesting a bilateral effect of MRgFUS thalamotomy on motor-related networks.

We found out that MRgFUS also had remote effects by modifying the balance of FC of ROIs within the CTC network but far away from the ablated lesion site. In this study, we investigated the FC changes of the primary motor cortex specifically located at the hand region (M1H) and found enhanced FC between the M1H and sensorimotor cortical regions encompassing precentral gyrus and postcentral gyrus and decreased FC between M1H and the cerebellum motor regions. Previous study reported that ET patients showed decreased FC between the primary motor cortex and the supplemental motor area, which are two key regions involved in motor planning and execution (30). Our finding may suggest that the MRgFUS thalamotomy restored the abnormal FC within sensorimotor network in ET. The increased FC in the sensorimotor network after thalamotomy treatment were also found in other rs-fMRI studies (26, 31–33).

Enhanced FC of left dentate nucleus (DN) was found in the sensorimotor cortical regions including bilateral precentral gyrus and supplemental motor area, as well as decreased FC were found in cerebellar motor area and brain stem after thalamotomy. The alterations in the FC of the DN following thalamotomy hold significant implications for our understanding of motor control and tremor modulation in ET patients. The DN, as a key structure within the cerebellum, plays a crucial role in coordinating motor commands and fine-tuning movements. Changes in FC patterns of the DN may reflect underlying shifts in the coordination of motor circuits, influencing the precision and timing of movement execution. Moreover, the DN is intricately connected to both cortical and subcortical regions involved in motor planning and execution (34, 35). Enhanced FC between the DN and sensorimotor cortical regions following thalamotomy may signify a reorganization of motor networks aimed at compensating for tremor-related deficits. Conversely, decreased FC in cerebellar motor areas and the brainstem may reflect a reduction in aberrant neural signals contributing to tremor generation.

Importantly, the DRTT (Dento-Rubro-Thalamic Tract) consists of both decussating and non-decussating fibers, which may differentially contribute to post-thalamotomy connectivity changes. Since left VIM ablation primarily targets decussating fibers, it is possible that increased information traffic through spared non-decussating fibers contributes to the observed FC alterations. This shift in network dynamics may represent a compensatory mechanism, wherein non-decussating pathways assume a greater role in motor signal transmission following disruption of the primary decussating pathways. Further studies incorporating tractography analyses are needed to delineate the relative contributions of these fiber populations to post-thalamotomy motor network reorganization.

Our association analysis between the FC changes of left VIM and the tremor improvement demonstrated significant positive correlation in the frontal lobe and cerebellum, as well as negative correlation in the sensorimotor cortical area and visual cortical area. The identified brain regions involved the motor, attention, visual and visuospatial function. Our findings may provide valuable insights into the neural mechanisms underlying thalamotomy treatment in ET. The significant positive correlation observed between FC changes of the VIM and tremor improvement, particularly in the frontal lobe and cerebellum, highlights the modulation of motor circuits following thalamotomy. The VIM, as a key node within the tremor network, regulates motor signals transmitted to the cortex and cerebellum. By modulating FC within these regions, thalamotomy may disrupt aberrant neural oscillations associated with tremor generation, leading to improved motor control and tremor reduction. The negative correlation observed in sensorimotor and visual cortical areas suggests a potential role of attentional and visual processing in tremor modulation post-thalamotomy. Attentional resources and visual feedback are integral components of motor control and coordination. Altered FC in these areas may reflect changes in attentional allocation and sensory feedback mechanisms following treatment, contributing to the attenuation of tremor symptoms. The involvement of brain regions associated with visuospatial function underscores the complexity of motor planning and coordination in ET. Thalamotomy-induced changes in FC within visuospatial networks may reflect the integration of visual information with motor output, facilitating more precise and coordinated movements post-treatment. Interestingly, the association observed in the M1 region was not localized to the hand area but rather positioned closer to the midline, suggesting that the FC changes may involve proximal or axial motor control rather than isolated hand movements. Given that thalamotomy for essential tremor (ET) often affects broader motor circuits, this finding may indicate a role of trunk or postural control mechanisms in tremor modulation.

Additionally, we found a positive correlation between tremor improvement after MRgFUS and the functional connectivity of the insula and temporal pole. The insula is involved in sensorimotor integration, interoception, and emotional regulation, all of which may contribute to tremor processing. Enhanced FC in this region post-treatment could reflect adaptive changes in how sensory and motor information is integrated, potentially improving motor control. The temporal pole, on the other hand, is implicated in higher-order associative functions, including visuomotor integration and emotional processing. Its involvement suggests that non-motor networks may also play a role in tremor improvement, possibly through changes in perception, motor awareness, or cognitive-emotional influences on movement control. These findings highlight the multifaceted neural mechanisms underlying MRgFUS-induced tremor reduction, extending beyond traditional motor pathways to include sensorimotor, cognitive, and emotional regulation networks.

### 4.1 Limitations

This study has several notable limitations. First, the small sample size of ET patients reduces statistical power and increases the risk of Type I and Type II errors. We carefully selected voxel-wise statistical thresholds (uncorrected *p* < 0.01, FDR-corrected *p* < 0.05) appropriate for the sample size, but the potential for false positives remains. Future studies with larger cohorts are needed to validate and generalize our findings.

The absence of healthy controls represents another significant limitation. Without comparative data, we cannot definitively determine if the observed reductions in VIM functional connectivity (FC) represent a restoration to normal levels or persistent disparities. Existing literature indicates ET patients typically exhibit increased VIM FC compared to healthy controls (29). While our findings suggest VIM MRgFUS treatment reversed this pattern, a direct comparison with a healthy control group would provide more conclusive evidence.

Our analysis of the ablated left VIM functional connectivity may raise concerns due to potential cell death or necrosis. However, FC analysis focuses on synchronized activity patterns across brain networks, not individual neuron integrity. The small necrosis core, covering only partial VIM regions after 1-year recovery, did not compromise our findings of significant FC disruptions in the left VIM sensorimotor cortical network.

A potential limitation of our study is the use of manually defined ROIs with specific coordinates, as opposed to standard atlas-based or segmentation-based methods. This approach was deliberately chosen to capture the precise treatment area in MRgFUS with maximal anatomical specificity relevant to our research questions. While atlas-based or segmentation-based techniques offer standardization, our coordinate-based method allowed for a more targeted analysis of the specific treatment volume. Future studies might benefit from comparing our coordinate-based approach with atlas-based or segmentation-based methods to validate the precision and reproducibility of our method.

The study's gender representation is limited, with only one female patient, potentially constraining our ability to assess sex-related FC changes. Although our analysis excluding the female subject showed no significant result variations, future research should employ larger, more balanced cohorts to control for potential sex differences.

Lastly, while our primary focus was tremor relief, the observed FC changes suggest potential cognitive implications. The lack of formal cognitive assessments limits our understanding of neuroplastic alterations' broader neurological impacts. Given the mixed reports in the literature regarding cognitive decline following FUS thalamotomy (36), future studies should incorporate standardized cognitive tests to comprehensively evaluate VIM thalamotomy's effects beyond tremor improvement.

## 5 Conclusion

Collectively, our findings of the altered FC in the CTC network and the association between the changed FC and tremor improvement may help us gain valuable insights into the development of targeted interventions aimed at restoring motor function and alleviating tremor symptoms in ET patients.

## Data Availability

The original contributions presented in the study are included in the article/Supplementary material, further inquiries can be directed to the corresponding authors.

## Supplementary material

Supplementary Figure 1 Significant associations between changes in right-hand tremor scores (pre-post) and left VIM functional connectivity (FC) changes (post-pre). **(A)** Brain clusters showing significant negative correlations. **(B)** Brain clusters showing significant positive correlations. **(C)** Scatter plots illustrating the negative correlations between right-hand tremor score changes and left VIM FC changes from significant clusters. **(D)** Scatter plots illustrating the positive correlations between right-hand tremor score changes and left VIM FC changes from significant clusters.

Supplementary Figure 2 Functional connectivity (FC) difference in the left VIM between pre- and post-1year treatment for subjects with N = 15 **(A)** and N = 14 **(B)**, after excluding one subject with low peak treatment temperature.

Supplementary Figure 3 Functional connectivity (FC) difference in the left M1H between pre- and post-1year treatment with different ROI sizes (radius): **(A)** r = 3 mm; **(B)** r = 5 mm; and **(C)** r = 7 mm.

Supplementary Table 1 Significant clusters from the association analysis between changes in left VIM FC and right hand tremor scores.
