# Supplementary figures and images for: MR-guided focused ultrasound thalamotomy modulates cerebello-thalamo-cortical tremor network in essential tremor patients
Source: Front Neurol. 2025 Apr 22;16:1526501. doi: 10.3389/fneur.2025.1526501 (PMC12053286; doi:10.3389/fneur.2025.1526501)

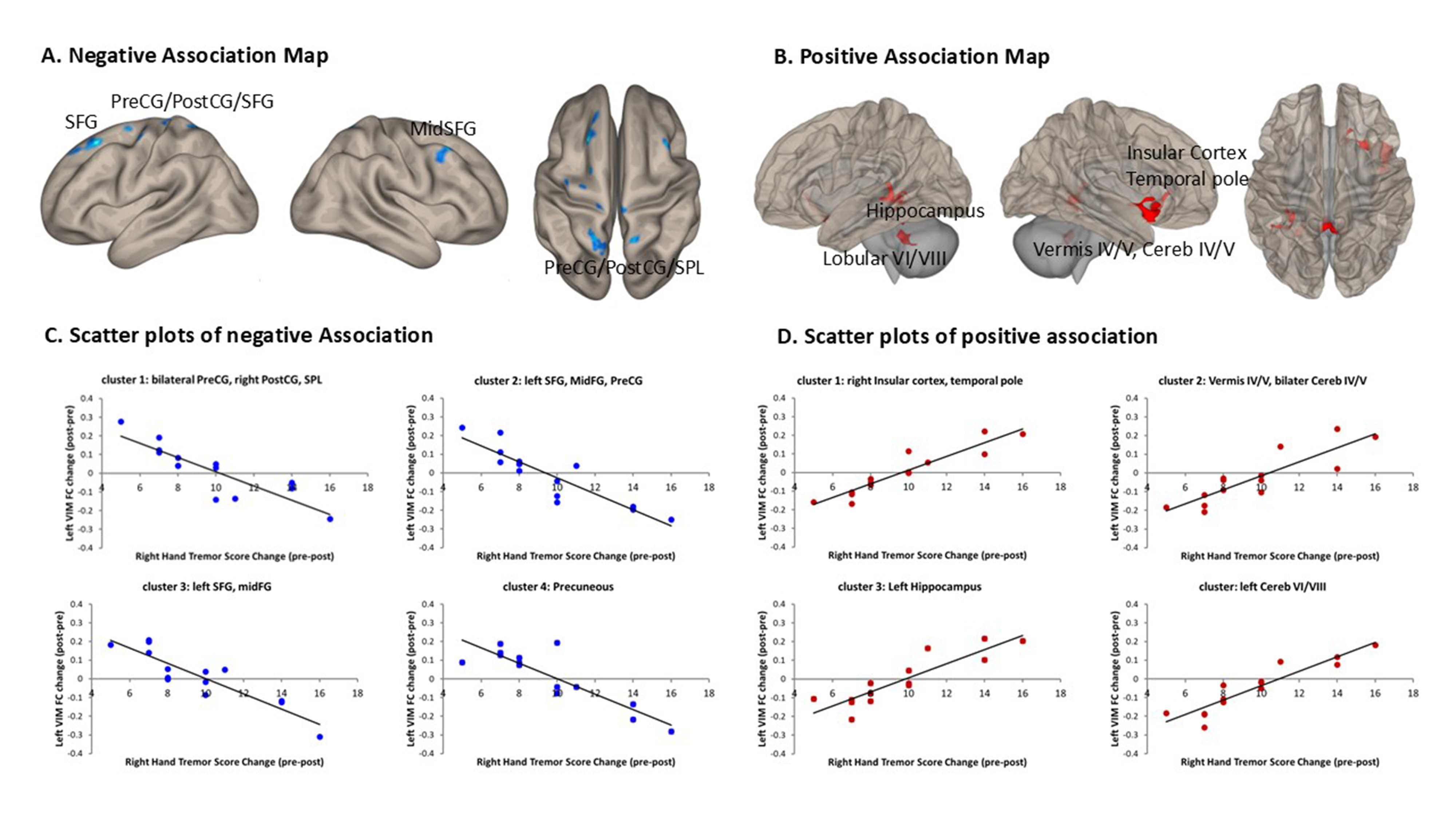

Supplement: Supplementary Figure 1 — Significant associations between changes in right-hand tremor scores (pre-post) and left VIM functional connectivity (FC) changes (post-pre). (A) Brain clusters showing significant negative correlations. (B) Brain clusters showing significant positive correlations. (C) Scatter plots illustrating the negative correlations between right-hand tremor score changes and left VIM FC changes from significant clusters. (D) Scatter plots illustrating the positive correlations between right-hand tremor score changes and left VIM FC changes from significant clusters. [file Image_1.jpeg]

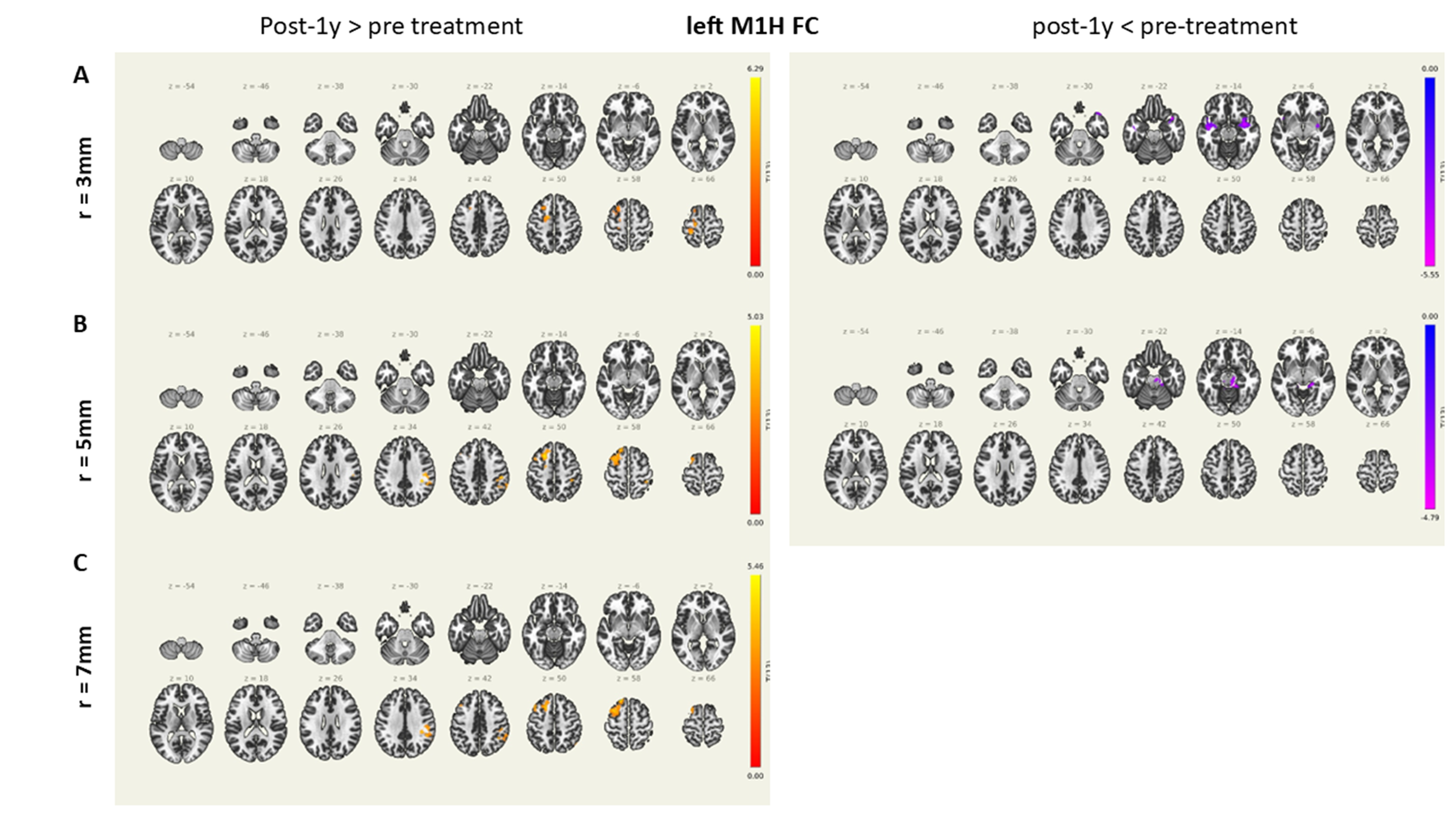

Supplement: Supplementary Figure 2 — Functional connectivity (FC) difference in the left VIM between pre- and post-1year treatment for subjects with N = 15 (A) and N = 14 (B), after excluding one subject with low peak treatment temperature. [file Image_2.png]

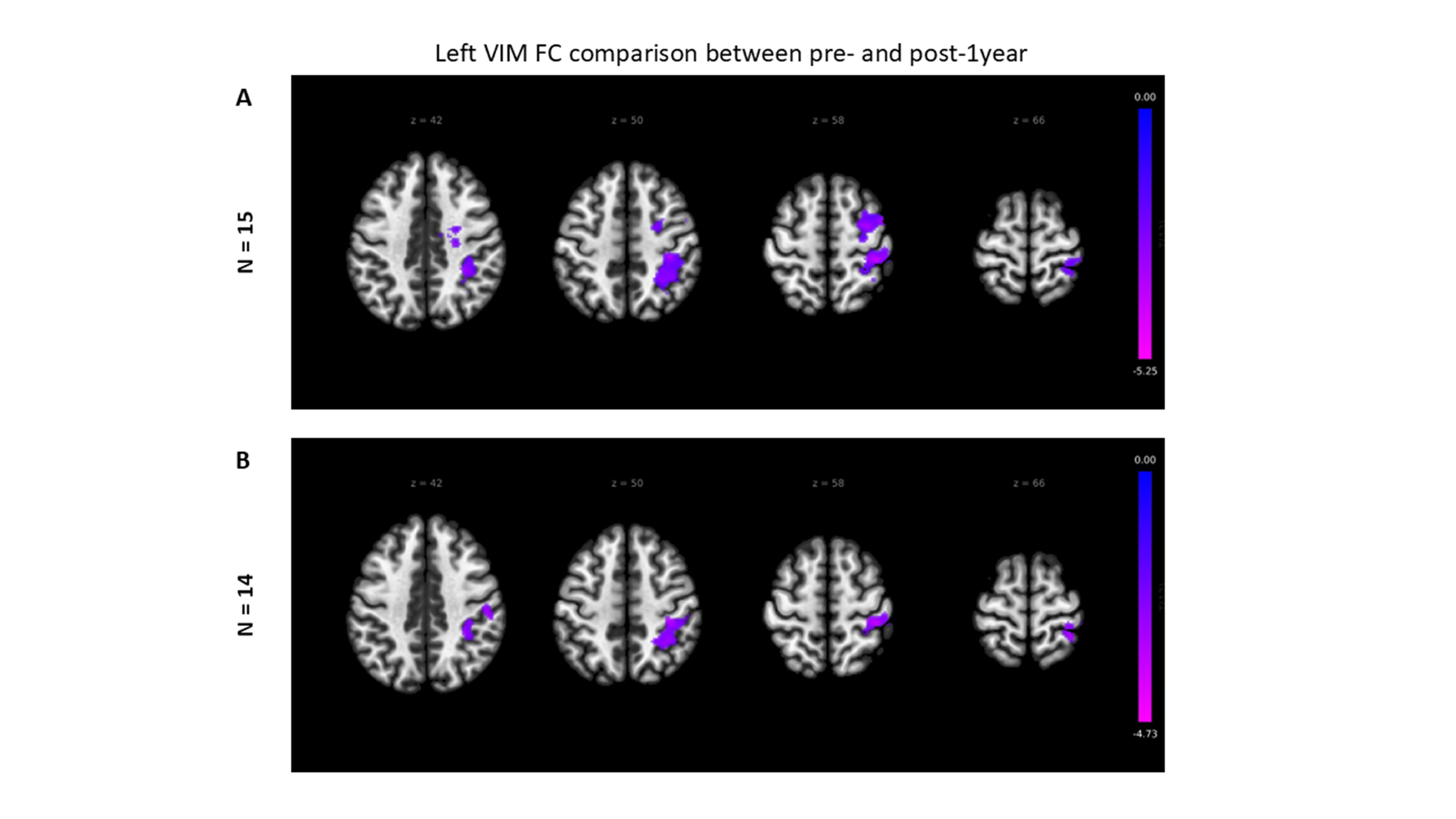

Supplement: Supplementary Figure 3 — Functional connectivity (FC) difference in the left M1H between pre- and post-1year treatment with different ROI sizes (radius): (A) r = 3 mm; (B) r = 5 mm; and (C) r = 7 mm. [file Image_3.png]
