# Supplementary material for: MR-guided focused ultrasound thalamotomy modulates cerebello-thalamo-cortical tremor network in essential tremor patients
Source: Front Neurol. 2025 Apr 22;16:1526501. doi: 10.3389/fneur.2025.1526501 (PMC12053286; doi:10.3389/fneur.2025.1526501)
Supplement: Supplementary Table 1 — Significant clusters from the association analysis between changes in left VIM FC and right hand tremor scores. [file Table_1.docx]

Supplemental Table 1: Signficant clusters from the association analysis between changes in left VIM FC and right hand tremor scores.
